# An in-vitro study of the timing between protamine sulfate administration and cardiotomy suction termination

**DOI:** 10.1051/ject/2025028

**Published:** 2025-12-17

**Authors:** Jennifer Gavin-Veyna, Carolyn Taylor Troester, Reid Homann, Samantha Varela, Amber Lickert, Scott C. Sanderson

**Affiliations:** 1 University of Nebraska Medical Center S 42nd & Emile St. Omaha NE 68191 USA

**Keywords:** Protamine, Heparin, Cardiopulmonary Bypass, Cardiotomy Suction

## Abstract

*Background*: During cardiopulmonary bypass (CPB), anticoagulation of the blood is the paramount responsibility of a perfusionist. The perfusionist should ensure the termination of cardiotomy suction at the onset of protamine sulfate (protamine) administration to prevent compromising the integrity of the extracorporeal circuit (AmSect. Standards and Guidelines for Perfusion Practice. 2023. https://www.amsect.org/Policy-Practice/AmSECTs-Standards-and-Guidelines). Although coagulopathy causes the largest mortality risk in adult CPB cases, standardization is not seen universally, and practice often varies between institutions (Stammers et al. Perfusion. 2001;16(3):189–198. https://doi.org/10.1177/026765910101600304; Jansa et al. Ann Thorac Surg. 2022;113(2):506–510. https://doi.org/10.1016/j.athoracsur.2021.04.059). *Methods*: Activated clotting times (ACTs) were measured in five swine subjects that were heparinized and placed on CPB for a total of approximately 6 h each. Samples of blood were drawn from the CPB circuit; ACTs were measured before the administration of protamine, after a protamine test dose (PTD), and after 1/3 of the full protamine dose had been introduced to each sample. Protamine dosing was determined by a 1:100 ratio of protamine to heparin. *Results*: 60 blood samples were included in the final analysis. The mean ACT after the PTD was 290.4 s (seconds), and 147.5 s after 1/3 of the full protamine dose. ACTs after the PTD decreased significantly by an average of 38.2% (*p* < 0.0001), and by 50.8% (*p* < 0.0001) after 1/3 of the full protamine dose was given. *Conclusion*: This investigation demonstrated an analysis of heparin reversal via protamine administration. The findings revealed that in the majority of samples, the PTD was sufficient to decrease the ACT below 480 s, the determined benchmark upon which CPB can be safely conducted. After 1/3 of the full protamine dose was given, nearly every sample’s ACT reached a value considered unsafe for bypass. The interpretation of the data suggests that there are significant grounds for advocating for a more disciplined approach to cardiotomy suction termination to preserve the integrity of the CPB circuit and to safely conduct CPB.

## Introduction

Patients undergoing open heart surgery require total anticoagulation, which is most often achieved using heparin and measured via ACT (in seconds) on a point-of-care device. Featured on the World Health Organization Model List of Essential Medications, heparin is an acidic polysaccharide that binds to antithrombin III, where it inhibits the formation of fibrin in blood by inactivating thrombin and factor Xa [1, 2]. Once the patient’s surgery has been completed and the patient has been weaned from cardiopulmonary bypass (CPB), the anticoagulation is reversed with the use of protamine. When protamine is administered, it antagonizes heparin by forming an inert salt with bound heparin molecules, which results in complete neutralization of heparin and reestablishes patient hemostasis [3, 4].

Protamine can be dosed based on the heparin concentration circulating in the patient, the total amount of heparin administered to the patient throughout CPB, or by using a fixed dose. For this study, the protamine dosing regimen was determined to be 1 mg protamine per 100 IU heparin administered in the loading dose, as this is a common technique used by clinicians to dose protamine [1, 5]. Despite the structured delivery of protamine, the reaction is not always consistent; while the goal of reestablishing hemostasis is reached, the rate at which this reaction occurs has a wide variance. Ten percent of the full protamine dose is often used as a “test dose,” as it will determine if the protamine causes hemodynamic instability [6]. Adverse effects of protamine include decreased systemic vascular resistance, pulmonary hypertension, and anaphylaxis [4]. The progress of protamine administration is tracked and announced by anesthesia periodically, indicating to the perfusionist when cardiotomy suction is to be terminated.

Cardiotomy suction is utilized in CPB to remove shed blood from the surgical field. This blood is then passed through a cardiotomy filter before returning to the venous reservoir for delivery back to the patient. The use of cardiotomy suction, like all of CPB, is dependent on the anticoagulation of the blood to prevent thrombus formation in the circuit, which inhibits the ability of CPB to continue. A point of emphasis in clinical perfusion is determining when to terminate the use of cardiotomy suction once protamine administration has begun. The goal of this timing is to maximize shed blood return while avoiding clotting within the CPB circuit and maintaining circuit integrity. Shed blood is of great concern for the surgical field as this indicates where additional suturing or hemostatic agents may be required; by leaving the cardiotomy suction on for a longer duration, this shed blood can be quickly returned to patient circulation via a cannula placed in a large arterial vessel.

CPB conduct and safety have been scrutinized and analyzed for over 45 years [7]. The American Society for Extracorporeal Technology (AmSECT), the governing body for cardiovascular perfusion and related services, has defined a standard of practice regarding this research. Standard 12.1 of *AmSECT’s Standards and Guidelines for Perfusion Practice* states, “Cardiotomy suction shall be discontinued at the onset of protamine administration to avoid clotting within the cardiopulmonary bypass circuit” [8]. Despite this standard being in place, other cardiotomy suction termination benchmarks remain. Some institutions terminate cardiotomy suction after the PTD, while others wait until 1/3 of the full protamine dose has been administered. Continuing cardiotomy suction during protamine administration maximizes the amount of chest cavity blood suction and shed blood return, but it significantly increases the risk of thrombus in the CPB circuit. On the contrary, ceasing the use of cardiotomy suction immediately after starting administration allows for maximum protection of the CPB circuit from clotting, but offers the least amount of shed blood return post-CPB. Due to the unpredictable reaction rate of protamine, this risk is difficult to quantify, and little evidence exists defining the risk this may impose on the patient. This research intends to determine whether the PTD or 1/3 of the full dose of protamine is the ideal time to terminate cardiotomy suction.

## Materials and methods

This project was performed on porcine subjects during the University of Nebraska Medical Center (UNMC) Clinical Perfusion In-Vivo Laboratory Series. This offered a readily available patient population and allowed the utilization of samples already necessary for the lab’s basic function. All animals used in this study received humane care in compliance with the “Guide for the Care and Use of Laboratory Animals.” The policies and procedures were conducted following Institutional Animal Care and Use Committee (IACUC) guidelines and approval (IACUC #17-073-12-FC, approval date 4/19/23), and all researcher participants completed both IACUC and UNMC institute-based training and testing. The In-Vivo Simulation Lab spanned six days and involved the use of a different test subject each day; however, data was only used from five of these days. On the initial day of testing, the data was skewed by miscalculated protamine dosing and was therefore omitted from the final data pool. For this research, the patients discussed shall be understood to be porcine subjects under standardized CPB conduct.

The administration of protamine is a significant event within cardiac surgery involving CPB; therefore, ensuring the accuracy of dosage and timing of administration is key. These two points were emphasized while conducting research to simulate the highest level of accuracy achievable in a simulated scenario. Additional accuracy was ensured by using only methods and devices determined as standards of practice by AmSECT. Hemochron Signature Elite HX1100 (International Technidyne, Edison, NJ, USA) devices and ACT+ cuvettes were used to measure the ACT of each sample at different levels of anticoagulation; each device was quality control tested per the manufacturer’s instructions for use manual [9].

Patient weight was measured using a scale by the UNMC laboratory staff and was provided at the beginning of each day. Once a central line was placed, a sample was drawn in a 3 milliliter (mL) syringe to determine a baseline ACT of the patient. Before cannulation, a loading dose of heparin was administered by the laboratory staff upon the surgeon’s request; this loading dose consisted of 400 International Units (IU) of heparin per kilogram weight of the patient. After the heparin had circulated for 3–5 minutes (min), another sample was drawn from the central line to determine the post-heparin ACT. Cannulation was performed, and CPB was initiated at normothermia once the ACT reached 480 s. 10,000 IU heparin was also added to the prime solution in the CPB circuit. Once on CPB, 3 mL syringes were used to draw 1–3 mL samples of arterial blood from the circuit manifold. Additional boluses of heparin were administered via the manifold to treat an ACT less than 480 s and samples were drawn at a minimum of 3 min after administration; the dosage and time of administration were recorded for each bolus. Boluses of normal saline were given as needed to maintain an adequate level in the CPB circuit reservoir.

After drawing each heparinized sample, a preprogrammed “Protamine Calculator” was used to administer an exact dose of protamine to the sample. This calculator was an Excel spreadsheet with functions embedded in order to calculate the precise amount of protamine to add to each sample. The weight of the test subjects was used to estimate blood volume. By factoring in the amount of heparin in the patient along with the size of the sample, the calculator determined the amount of heparin present in each sample. A 1:1 ratio was then used to calculate the full dose of protamine needed to reverse the heparin in the sample (1 milligram [mg] protamine per 100 IU heparin). This value was then used to determine the amount of protamine needed for a PTD and for 1/3 of the full protamine dose. The Excel spreadsheet minimized human error within the procedure and allowed the team to organize any additional variables.

The protamine concentration provided by the UNMC pharmacy was 50 mg/5 mL, necessitating dilution of the protamine for accurate dosing. Dilution was also required due to the small size of the samples. The protamine was diluted using 99 mL of 0.9% normal saline and 1 mL of protamine, resulting in a final concentration of 0.5 mg/mL. Once diluted, the protamine could be added to the samples in precise doses to accurately reverse the heparin in the sample. The doses added to the samples were 10% of the full protamine dose and 1/3 of the full protamine dose to simulate the aforementioned common benchmarks for cardiotomy suction termination.

The process of drawing samples, measuring ACTs, and administering protamine for varying heparin concentrations was well documented and consistent throughout testing. Each heparinized sample was tested for an initial ACT, after PTD administration into the sample, and then after 1/3 of the full protamine dose was administered into the sample. These stages were chosen as they reflected a common timeline of events for the total reversal of heparin. ACTs after the full protamine dose were not measured to reduce the number of ACT cartridges; additionally, it was inferred that the heparin would be fully reversed with the full protamine dosing.

Two Hemochron devices were used per the manufacturer’s guidelines in the measurement of the samples to ensure rapid testing and minimal clotting within each sample syringe after it was drawn (Hemochron manual). Hemochron #1 was used for baseline ACT and post-heparin ACT measurements, and Hemochron #2 was used for the PTD and 1/3 of the full dose protamine administration. Hemochron #2 was chosen for both the PTD and 1/3 dose of protamine due to the significant time required to complete the post-heparin ACT in each sample. Samples were then discarded in a biohazard waste container. Each Hemochron device was cleaned and powered off for the following day. All storage, quality control, and usage were followed per the manufacturer’s guidelines and instructions for use. Additionally, all heparin and protamine were stored in a locked cabinet to ensure no mishandling or improper use of drugs.

The sample size was determined by the time on bypass for each patient; each test subject was on CPB for a total of approximately 6 h. Due to the amount of time on CPB, the N number was 60 samples with three different measurements made on each sample. This number does not include the baseline ACT taken at the beginning of each day, as this was not a testable sample due to the lack of heparin in the patient before full-dose heparin was administered.

Data was gathered concurrently with ACT testing to ensure accuracy. The same data points were collected for each sample, and the data were discussed by each group member before transferring recording responsibility. All four group members recorded data collectively and used identical sampling and recording techniques. The emphasized points of data recording were as follows: date of collection, sample number, weight of the test subject, sample volume, baseline ACT, heparin doses given (designated by loading dose, included in prime and additional boluses), post-heparin ACT, total volume of the PTD, ACT after the PTD, total volume of 1/3 full dose protamine, ACT after 1/3 full dose protamine, and the full dose of protamine which would reverse the heparin in the sample.

A baseline ACT measurement was collected every day before heparin administration, and a second ACT was drawn after the loading dose of heparin was given before going on CPB. Another ACT was drawn after initiation of CPB, since additional heparin was added to the CPB circuit. ACTs were then drawn periodically throughout CPB; all data were recorded in chronological order.

Statistical analysis was conducted using a total of 60 samples collected over five days. A linear mixed model with a random effect to account for the correlation within blood samples and a fixed effect for varying protamine doses was used to compare the ACT from each sample. Model-adjusted means and standard errors (SEs) were used to describe the ACT at each dose level. Analyses were performed using SAS, Version 9.4.

## Results

Three ACTs (5%) measured less than 320 s after the heparin loading dose and were excluded from the analysis. “Out of range” ACTs were measured to be greater than 1005 s; these outliers were included in the data analysis. 51.7% of ACTs were out of range after heparin administration, and 3.3% remained out of range after the PTD. Only one sample (1.7%) was still out of range after 1/3 of the full dose of protamine. These outliers may be attributed to testing or human error, physiologic variability amongst test subjects, or using a mathematical approximation to calculate the estimated blood volume.

After administration of the PTD, 95% of the ACTs were less than 480 s, and 96.7% of the ACTs were under 480 s after 1/3 of the full protamine dose was given. Additionally, 58.3% of the samples had ACTs less than 300 s after the PTD was given, and 88.3% of the samples had ACTs below 300 s after 1/3 of the full protamine dose was added. These findings verify the validity of the study regardless of whether the initial ACT may have been higher than anticipated. Predictably heparin reversal was seen despite the initial ACT increase.

**Figure 1:**
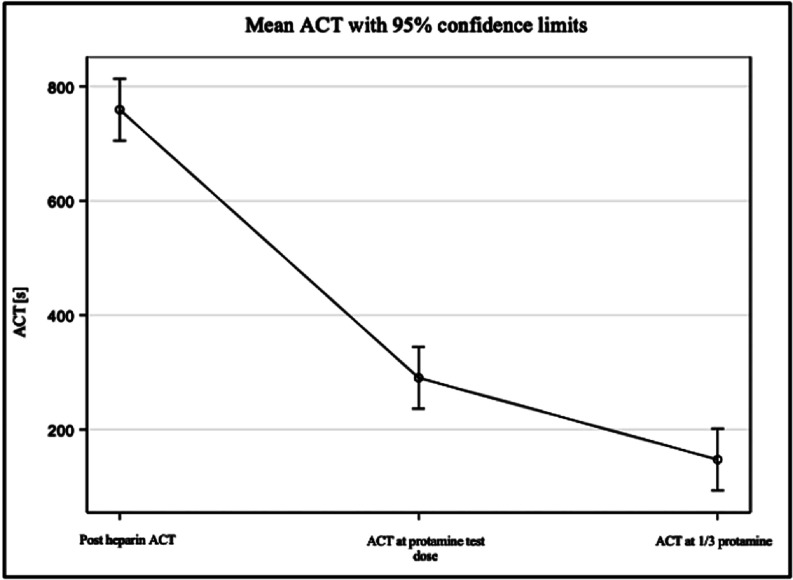
Nonlinear decrease in average ACT with 95% confidence limits.

**Figure 2:**
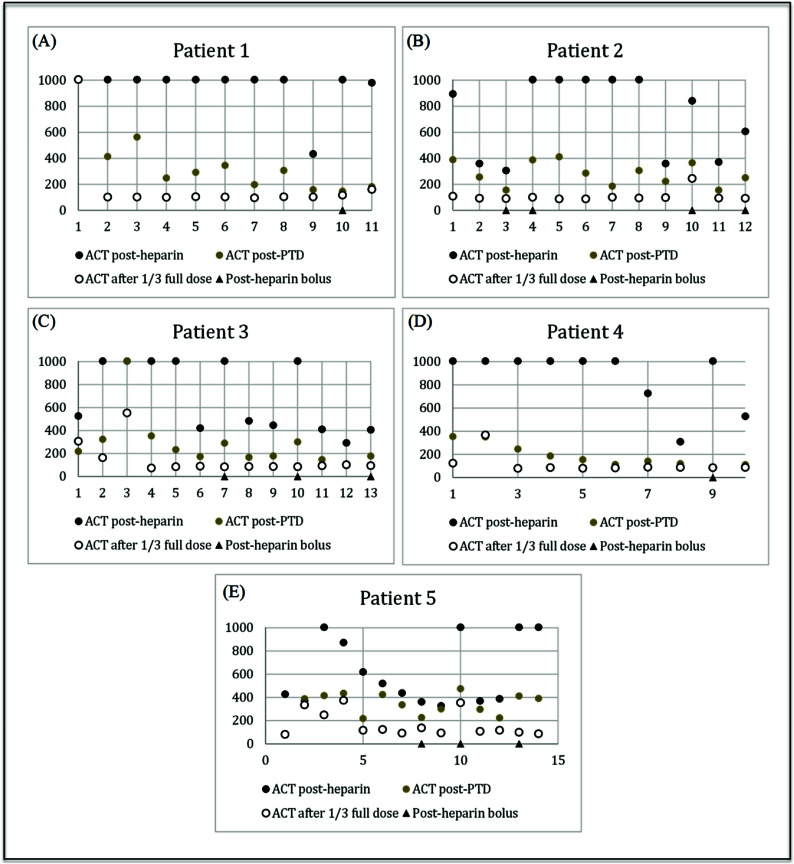
(a)–(e) ACT values for patients 1–5 after heparin, after PTD, and after 1/3 full dose protamine; triangles identify ACT results after a heparin bolus was administered.

As seen in Table 1, on average, the initial post-heparin ACT was 759.45 s. After the PTD, the mean ACT was 290.35 s with a standard deviation of ±173.02 s. The mean ACT measured 147.53 s after 1/3 of the full protamine dose with a standard deviation of ±146.05 s. ACTs after the PTD decreased by 38.2%, and by 50.8% after 1/3 of the protamine dose. A *p*-value less than 0.05 was considered to be statistically significant. As seen in Table 1, upon PTD administration the decrease in ACT between the post-heparin dose and the PTD was found to be statistically significant (*p* < 0.0001). The p-value between the PTD and 1/3 of the full protamine dose was also less than 0.0001, making these findings statistically significant as well. Figure 1 is a visual representation of the nonlinear decrease in mean ACTs with 95% confidence. A graphical representation of the ACTs post-heparin, after PTD and after 1/3 of the full protamine dose is depicted in Figure 2; each figure shows the ACTs as they were drawn in chronological order per day.

**Table 1:** Average ACT at different checkpoints (post-heparin, PTD, and 1/3 full protamine dose) with standard deviation and nonlinear decrease.

ACT Checkpoint	Model adjusted mean (s)	Decrease in ACT (%)	*p*-Value
ACT post-heparin	759.45	N/A	N/A
ACT after PTD	290.35 (±173.02)	61.77	<0.0001
ACT after 1/3 full dose protamine	147.53 (±146.05)	49.19	<0.0001

## Discussion

The porcine test subjects were all under general anesthesia and received total anticoagulation for CPB, and the samples of blood collected were exposed to the entire CPB circuit to simulate as close to a real-life environment as possible in vitro. Blood was circulated and returned to the reservoir via cardiotomy suction tubing as it would during a typical CPB case, ensuring the accuracy of data by simulating the blood’s exposure to realistic surfaces and the implied hemostatic repercussions. The in vitro design of this study eliminated the risk of the test subjects developing a sudden protamine reaction since the reversal agent was not administered to the patient and was instead micro-pipetted into syringes. Heparin neutralization via a 1:100 dosing method for protamine may have also reduced any chances of accidental protamine overdose. Excessive protamine administration is known to have the potential to cause post-operative bleeding and an increased risk of blood transfusions due to the anticoagulant effect of protamine in the absence of heparin to bind to [5, 10]. Unbound protamine has been shown to decrease thrombin generation, impair activation of factors VII and V, potentiate fibrinolysis, and inhibit platelet function [3, 10]. In one study, patients who had received excessive amounts of protamine exhibited raised ACT, INR, and aPTT values and required more transfusions compared to those who did not receive as much protamine [11].

This study demonstrated a reasonably conclusive yet limited analysis of heparin reversal via protamine and the need for stricter adherence to perfusion standards and protocols within the operating room setting by all members of the cardiac team. In the majority of samples, it was found that the PTD was sufficient to reverse heparin levels enough to compromise circuit integrity and provide unsafe conditions for CPB. After 1/3 of the full protamine dose was administered, nearly all ACTs had dropped to a value considered unsafe for CPB. These findings reinforce the need for following standards of practice for cardiotomy suction termination in order to maintain circuit integrity and thus safe CPB conduct.

Furthermore, the significance of the data collected in this study complements vast evidence for disciplined protamine administration and cardiotomy suction termination. Jansa and colleagues formulated very similar conclusions with extremely complementary results. Both studies indicated that the PTD yielded a majority of ACT results that are deemed unsafe for CPB circuit integrity. After reviewing the results of similar studies and the presented data, the authors hypothesize that protamine administration does not have a linear relationship with ACT values. Numerous studies have shown a highly significant decrease in ACT values after the PTD. The continuation of protamine administration can have a nonlinear and/or plateau effect on ACT values. This visualized non-linear relationship with protamine dosing makes ACT value prediction difficult. The presented data, along with numerous complementary studies, promote the clinically based suggestion of instituting cardiotomy suction termination as set by current guidelines, with the implementation of such practices within institutional protocols as a standard.

## Data Availability

All available data are incorporated into the article.
